# Thermal Expansion Behavior of Co-Spray Formed Al-20Si/7075 Bimetallic Gradient Alloy

**DOI:** 10.3390/ma14154100

**Published:** 2021-07-23

**Authors:** Lei Yu, Sida Jiang, Fuyang Cao, Hongxian Shen, Lunyong Zhang, Xu Gu, Heqian Song, Jianfei Sun

**Affiliations:** 1 School of Materials Science and Engineering, Harbin Institute of Technology, Harbin 150001, China; yulei605@163.com (L.Y.); hitshenhongxian@163.com (H.S.); allen.zhang.ly@gmail.com (L.Z.); xugu_hit@outlook.com (X.G.); songheqian_hit@163.com (H.S.); 2 Space Environment Simulation Research Infrastructure, Harbin Institute of Technology, Harbin 150001, China

**Keywords:** thermal expansion, functionally graded materials, Al-20Si/7075

## Abstract

Bimetallic gradient alloys have attracted research attention recently due to their potential applications in the aerospace and automobile industries. In this study, Al-20Si/7075 bimetallic gradient alloys were successfully manufactured by co-spray forming and the roll process. We investigated the thermal expansion behavior of the gradient alloy. It was found that the coefficients of thermal expansion increased with silicon content and increased temperature, reaching the highest point at 573 K, after which they decreased on account of the relaxation of residual thermal stress and the silicon desolvation from the supersaturated aluminum phase. The measured thermal expansion coefficient can be roughly predicted through the traditional theoretical models. Our results revealed the thermal expansion behavior of Al-20Si/7075 bimetallic gradient alloys and would improve the development of new type aluminum–silicon alloy for electronic packaging.

## 1. Introduction

In the last two decades, aluminum matrix composites have been widely applied in the field of scientific research and manufacturing on account of their excellent performance characteristics including low density and high strength, good elevated-temperature performance, and good friction performance [1,2]. Functionally graded materials (FGMs) have strong application prospects [3,4], as they have the capability to customize materials for specific applications, whose properties change with spatial coordinates [5,6]. As an initial point of failure, the distinct interfaces in composite materials can be eliminated [7]. In this case, a gradient interface takes the place of the distinct interface, resulting in a smooth transition area located between the materials that form the two sides. Li et al. [8] successfully prepared Ti-6Al-4V/SS316 functionally graded materials by LMD to combine the good mechanical and metallurgical properties of titanium alloys with the excellent weldability of stainless steel. Carroll et al. [3] completed the preparation and evaluation of SS304/IN625 functionally graded materials manufactured by DED additive manufacturing, which are useful in applications that require both strength and corrosion resistance at elevated temperatures. Cui et al. [9] studied graded tool steel for the micro cold forming tools manufactured by co-spray forming, and this new graded tool steel adapted the required strength of a material while retaining the desired abrasive wear resistance.

The 7075 alloy is extremely common and applicable in many fields because of its excellent mechanical performance such as high-specific strength, good plastic deformation, and good low temperature strength [10,11,12]. Similarly, Al-20Si alloy is widely applied in the aerospace and automobile industries, among others, because of its excellent wear resistance and thermophysical properties [2,13,14]. Therefore, functionally graded materials made from these two aluminum alloys may benefit from high strength, good friction performance, and outstanding thermophysical properties, so they were utilized in versatile cases, in particular electronic packaging. We have successfully manufactured several bimetal gradient plates with the composition of 7075 and Al-20Si alloy by the co-spray forming technique [15].

As for an electronic packaging material, thermal expansion is a critical property necessary to be evaluated because electric devices often produce a large amount of heat during working and thus induce strong temperature change in the packaging shell. Therefore, small thermal expansion is desired to avoid mechanical failure during the device working cycles. Researchers have proposed many models to analyze the thermal expansion coefficient (CTE) [16,17,18,19,20]. However, the thermal expansion properties of 7075/Al-20Si bimetallic gradient alloy are still unclear. In this work, the bimetallic gradient plates made with 7075 and Al-20Si alloy were prepared by the co-spray technique and densified by the rolling process. Their thermal expansion behaviors and relevant impacting factors were studied in order to shed new light on the development of electronic packaging materials based on aluminum–silicon alloy.

## 2. Experimental Details

### 2.1. Sample Preparation

**Figure 1:**
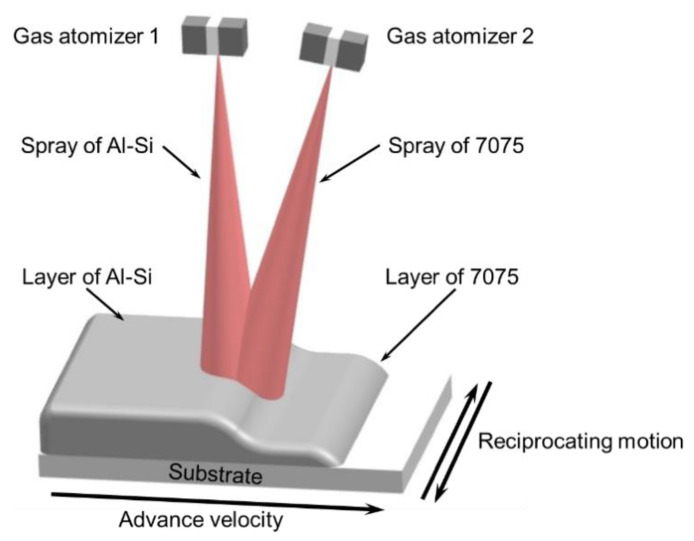
Diagram of the co-spray forming process principle.

The Al-20Si and 7075 alloys were atomized by two atomizers of a co-spray forming system, as shown in Figure 1. Instead of scanning by atomizers, the substrate was scanned in the transverse direction and at the same time translated horizontally through the two sprays. As the substrate performed a cyclical reciprocating motion, the droplets in the spray cone of 7075 alloy were deposited on the substrate first and formed the lower layer of the deposit. Following that, the droplets in the spray cone of Al-20Si alloy were deposited over the lower layer of the deposit and formed the upper layer.

**Figure 2:**
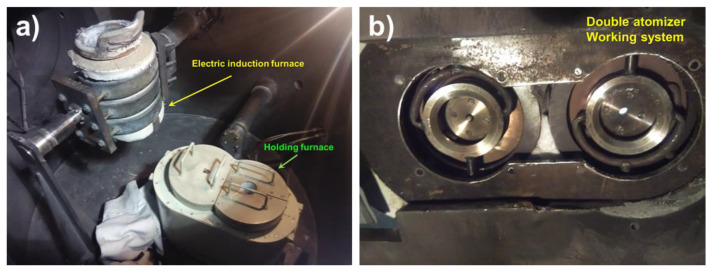
Modified test equipment for co-spray forming: (**a**) Smelting and insulation equipment, (**b**) double atomizer working system.

**Figure 3:**
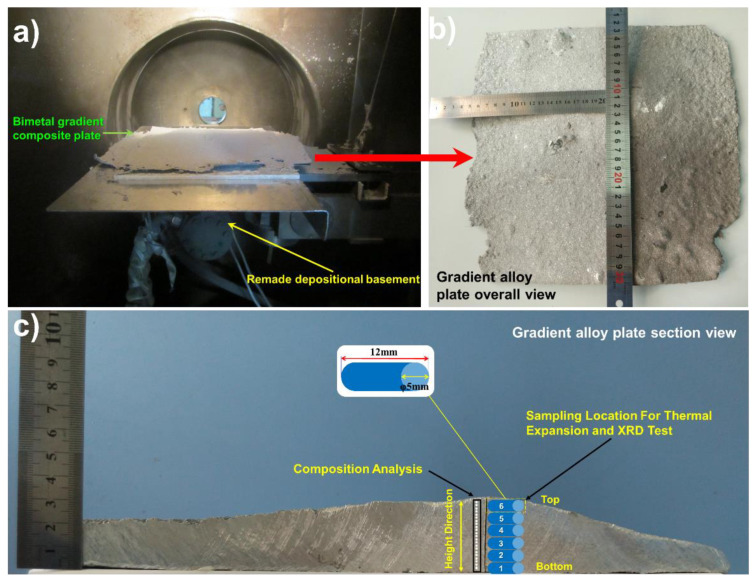
Bimetallic gradient composite plate and sampling location: (**a**) bimetallic gradient composite plate and depositional basement, (**b**) gradient alloy plate overall view, (**c**) section view and the sampling location for the test.

Co-spray forming experiments were performed by an original spray forming equipment that was specially modified for this experiment, as shown in Figure 2. The original single-station holding furnace was replaced with a double-station resistance furnace. The left side is responsible for insulation of the Al-20Si alloy, while the right side is responsible for heating and insulation of the 7075 alloy. The double atomizer working system had two atomizers that were independent of each other, and these were responsible for atomizing the Al-20Si and 7075 alloys. According to a previous study [15], the shape is mainly affected by the trajectory of the depositional basement, and the composition distribution of the deposited plate is affected by the center distance of the two atomizers, depositional basement scanning period, and advance velocity. Therefore, a normalized jaggies scanning mode which could be obtained by the remade depositional basement illustrated in Figure 3a was considered favorable for the formation of a flatter and more uniform deposited plate.

The composition of the 7075 alloy is listed in Table 1. The experimental parameters were set as follows. The melting temperature of the two alloys was set as 1123 K, which was the operating temperature used for spray forming as well. Then, the two melts passed through the pouring nozzles at the bottom of their respective tundishes and were atomized by means of high-speed nitrogen gas generated by two double-layer gas atomizers. The distance between the centers of the two different spray cones was set to 50 mm, the forward speed of the substrate was set to 1.5 mm s^−1^, the oscillation cycle was set to 1 s, and the stroke of substrate was set to 200 mm. The distance of atomizing deposition was set to 500 mm, and the outlet pressure of the atomizer was set to 1.2 MPa. Then, the bimetallic gradient alloy was densified by a specific rolling process with the temperature set to 783 K, and the alloy samples were rolled with a reduction of 10–15% for one pass. The total reduction in thickness was 50%.

### 2.2. Characterization of Structure and Physical Properties

As illustrated in Figure 3b, to verify the gradient characteristics of element distribution, the bimetallic gradient alloy samples with the center height of 30 mm were equally cut into 30 regions from top to bottom in the vertical direction. These 30 specimens for SEM and EDS were prepared by successive mechanical polishing with abrasive paper up to 2000 mesh (Mingniu Abrasive Materials Co., Ltd., Foshan, China). Then, these 30 specimens were polished with diamond paste. Afterwards, the 30 specimens were corroded with a sodium hydroxide solution in which the proportion of sodium hydroxide to water was 1:10. The microstructures of these 30 samples were observed through FEI Helios NanoLab 600 DualBeam FIB/SEM (SEM, Field Electron and Ion Company, Hillsboro, OR, USA), and the elements content was detected using an energy-dispersive spectroscopy detector (EDS, Field Electron and Ion Company, Hillsboro, OR, USA) by a plane scanning model. The phases were characterized using a Rigaku D/max-RB X-ray diffractometer (XRD) with monochromatic Co Ka radiation (XRD, Rigaku, Tokyo, Japan). XRD tests were carried out on six specimens obtained from six layers of bimetal gradient alloy samples, as shown in Figure 3c.

To explore the thermal expansion property of the material, according to the specified test sample size, the entire alloy ingot with a height of 30 mm was divided into six layers of the same thickness from bottom to top, and the first layer was the bottom layer (Figure 3c). The test samples were taken from each layer and made into specimens with a diameter of 5 mm and a length of 12 mm, so that the CTE of each layer was tested. The specimens prepared for CTE measurement were heated from room temperature to 673 K, and the heating rate was set to 5 K min^−1^ using a NETZSCH DIL 402SU dilatometer. To avoid the onset phenomena, the initial temperature point was set to 275 K. The argon gas filled the entire test chamber to provide a protective atmosphere and was circulated internally at a flow rate of 82 mL min^−1^ throughout the testing process. The length change of the sample was transmitted to the length measuring system by a push rod placed on the sample. Furthermore, in order to make the surfaces on both sides of the specimen parallel, the top and bottom of the specimen were polished in advance. The relationship between the bulk, shear, Young modulus, and temperature for the composite matrix was simulated by the JMatPro software.

## 3. Results and Discussion

### 3.1. Microstructure of Bimetal Materials

**Figure 4:**
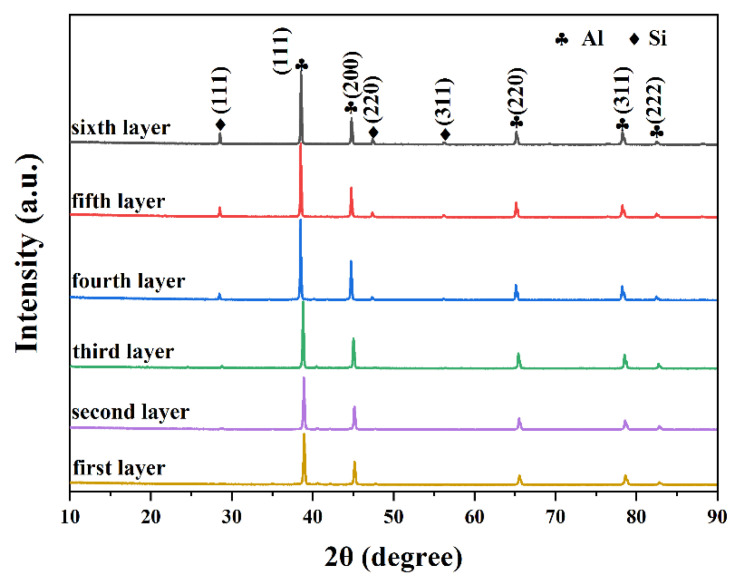
XRD results for each layer of gradient composite alloy.

**Figure 5:**
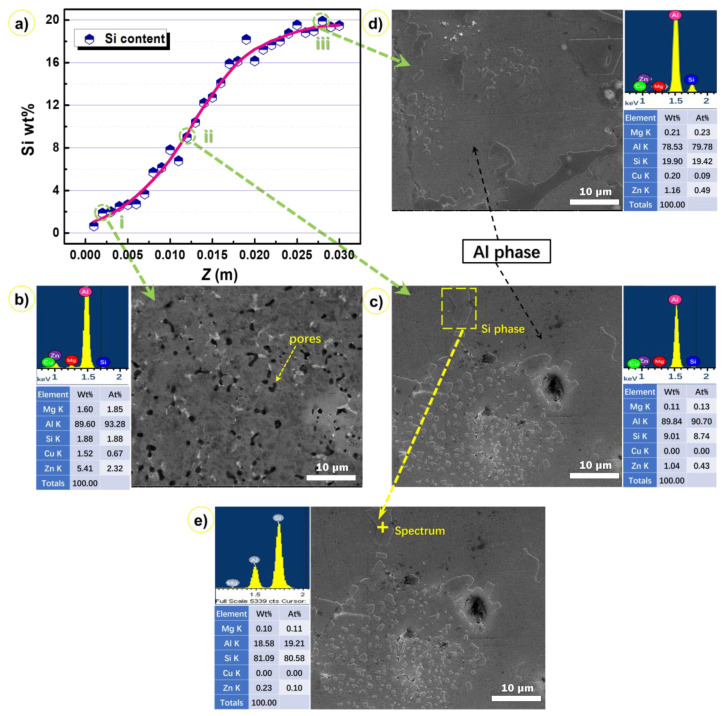
Silicon content and microstructures in the bimetallic gradient alloy. (**a**) Variation of silicon content in the 30 regions sampled, (**b**) microstructure and EDS of the lower region, (**c**) microstructure and EDS of the middle region, (**d**) microstructure and EDS of the upper region (the EDS data are obtained by an area scanning model), and (**e**) EDS spectrum of the rich-silicon phase.

Figure 4 gives out the XRD patterns of six samples cut from the six layers of bimetallic gradient alloy, as shown in Figure 3c. It was shown that all the samples basically contained only two phases, namely, Si phase and Al phase. According to the EDS results in Figure 5, the composition of Si phase is almost pure silicon. The crystal structure of Al phase is similar to that of pure aluminum; however, it should be noted that this phase contains the minor elements Zn, Mg, and Cu because these elements were not probed in this Si phase (Figure 5e). This might be a result of the high cooling rate of the spray forming process, over 10^3^ K s^−1^. The rapid cooling rate in the spray forming process makes the precipitation of Zn, Mg, and Cu difficult. Therefore, the associated alloying phase cannot precipitate. As shown in Figure 4, it can be found that the change of sampling position does not affect the phase composition of the gradient alloy, and no new phase is generated. The only difference is that the peak strength and so the content of the Si phase increases with the sample position from bottom to top.

Figure 5 shows the Si content probed by EDS at the 30 regions from the bottom to the top of the specimen. The Si content increased smoothly from the bottom to the top, confirming the composition gradient feature of the samples. This composition evolution was accompanied with a microstructure evolution that can be seen from the inset pictures in Figure 5, which show the typical microstructures of three selected regions noted by dashed circles in Figure 5a (located at the bottom (i), middle (ii), and top (iii) of the sample).

Figure 5b–d show SEM microstructure images of the Si phase distributed on the Al phase matrix, which are continuous. In terms of morphology, it can be divided into two types: one is the nearly spherical particle and the other is the lath-shaped silicon phase, as indicated by the point scanning EDS data shown in Figure 5e. The microstructure of the spray-formed alloy consisted of primary Si particles, and no Al-Si eutectic was found. During the cooling step of the atomization process, a large number of fine semi-solid primary Si particles emerged in the droplets before depositing on the substrate. The droplets impinge the substrate with a high speed and, as a result, they were melted again due to the latent heat of crystallization of the surrounding areas. During this process, the large primary Si particles can undergo cracking and fracture. Therefore, the fractured Si phase can lead to an increase in the nucleation sites. In the following solidification step, which displays a lower cooling rate, there was enough time for the Si atoms to diffuse toward the primary Si that had previously formed. The small black regions in Figure 5b are pores that are inevitable in any material processed through spray forming; indeed, the small pores that occurred in co-spray formed alloy are dependent on the processing conditions. On the basis of the study by Raghukiran et al. [21], atomized gas was involved in deposited billet during spray forming, facilitating the formation of spherical pores, while the formation of the irregular pores was due to the gap between spray droplets. The number of pores at the bottom of the deposit is more than that at the top, which may be due to the fact that the heat brought by the metal powder particles cannot be dispersed in time during the continuous growth of the deposit, leading to the remelting and re-solidification. Therefore, the shrinkage cavity defect formed in the cooling process, which is also one of the reasons for the formation of pores in the deposit.

**Figure 6:**
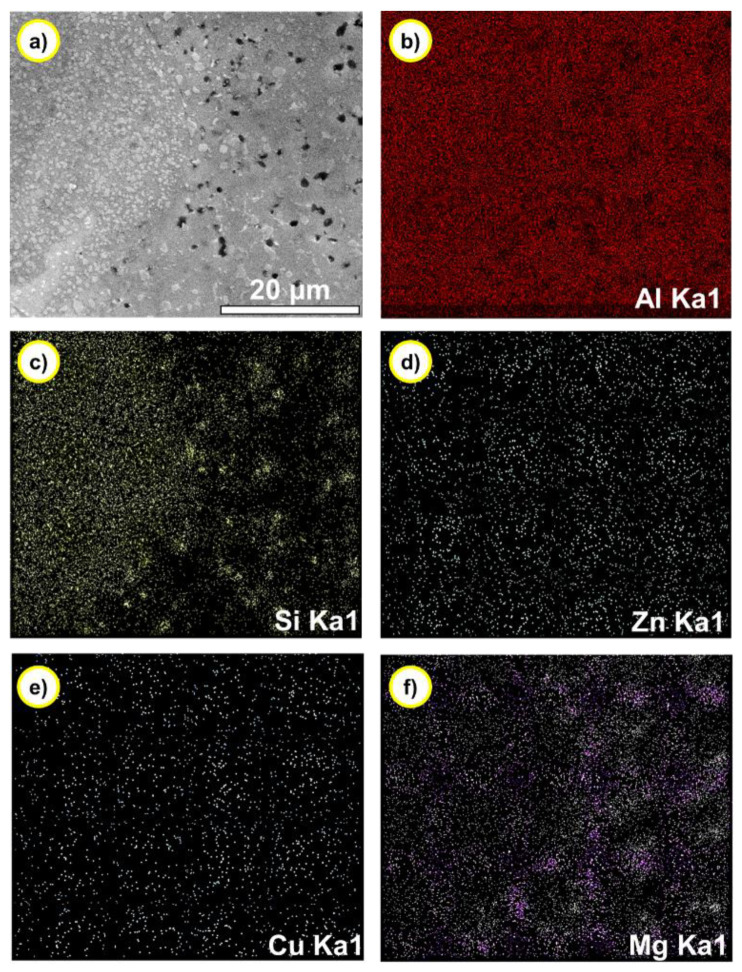
The distribution of elements in gradient composition alloy tested by plane scan of EDS. (**a**) Microstructure, (**b**) Al, (**c**) Si, (**d**) Zn, (**e**) Cu, and (**f**) Mg.

Figure 6 shows the distribution of elements in gradient composition alloy detected by the plane scan model of EDS. As shown in Figure 6b, combined with the XRD analysis in Figure 4, it can be found that the aluminum phase is the matrix phase of the gradient alloy and presents a continuous distribution. On the other hand, the distribution of silicon elements in Figure 6 also proves that the primary silicon phase is uniformly distributed on the aluminum matrix phase. The distribution of Mg, Zn, and Cu elements in Figure 6 also indicates the presence of 7075 alloy. This is consistent with the previous XRD and EDS analysis results.

### 3.2. Thermal Expansion Behaviors

**Figure 7:**
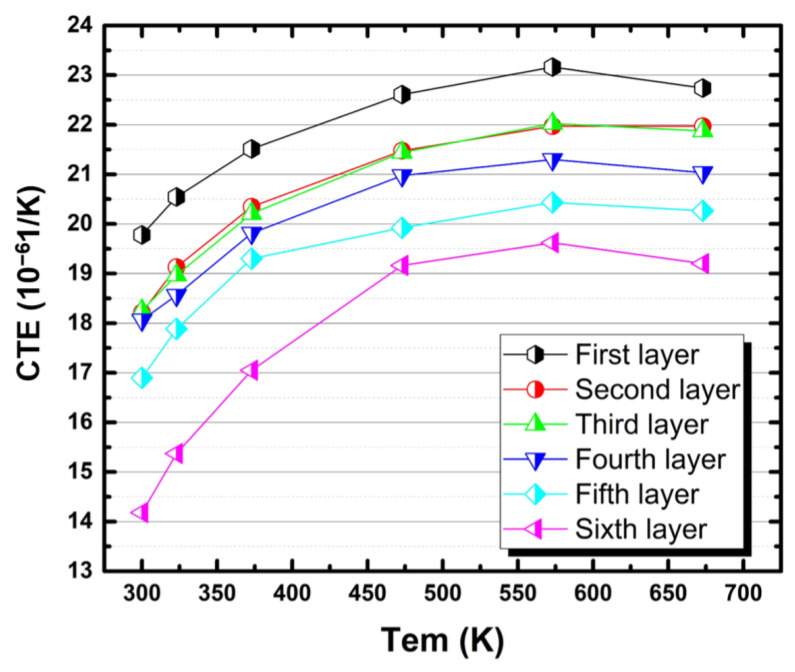
The CTE of different layers in terms of height of the bimetallic gradient composite alloy.

CTE is expressed as the change in dimension as a function of temperature [20]. In general, the average linear CTE (*α*) is acquired based on the following formula:

(1) $$\alpha_{t_{1},t_{2}}=\frac{L_{2}-L_{1}}{L_{0}\Delta T}=\frac{\Delta L}{L_{0}\Delta T}$$

where *L*_0_ represents the sample length at 303 K, and *L*_1_ and *L*_2_ represent the length corresponding to temperature *t*_1_ and *t*_2_, respectively. Figure 7 indicates the variation in CTE with temperature for different layers of the samples, and all curves show a similar trend. The CTE in each layer first increased with temperature to about 573 K; then, it tended to decrease as the temperature increased further. The CTE reduced with the increase of layer number. In other words, the increased silicon content reduced the CTE of the samples at a given temperature.

At the atomic scale, thermal expansion is caused by a change in the mean space between atoms. When the alloy is in the process of continuous heating, the silicon phase of the diamond structure is continuously precipitated from the matrix [18], in which the content of the silicon phase also continues to decrease. The lattice structure of aluminum is face-centered cubic, and there are four atoms in a cell, while in a face-centered cubic unit cell composed of aluminum and silicon atoms, there are four more silicon atoms, which are located at the mid-point of the four spatial diagonals [22]. In an Al-20Si supersaturated solid solution, Si atoms that are in excess tend to be located in the face center of the Al lattice [23]. The occurrence of precipitation makes silicon have a larger atomic volume than aluminum in a solid solution, so that the CTE increases significantly [24,25]. When the temperature exceeds 573 K, the silicon dissolves into the matrix, leading to a reduction in CTE.

The mechanism of thermal expansion for multiphase metal matrix composites is also applicable to this study. Therefore, the relationship between bulk, shear, Young modulus, and temperature for the composite matrix was simulated, and the Al:Si ratio of the first layer was defined as the matrix component. For different layers, these parameters were calculated by the ratio of the matrix and Si. The matrix does not deform plasticity at low temperature, and the expansion of the alloy is affected by the expansion of the matrix and the expansion of the Si phase together. As it was heated to a higher temperature, the matrix may deform plastically due to a lower yield strength compared with the thermal stress. In this case, the expansion was caused by the matrix, Si particles, and the plastic deformation of the matrix. However, the expansion of aluminum and silicon can also counteract some of this plastic deformation [26]. Thus, during the high-temperature stage, the increase of CTE continues, but the rate of thermal expansion growth is obviously reduced compared with that of the low-temperature stage.

The CTE of composites is affected by the plasticity of the matrix and the internal structure of composites [26,27,28]. The calculation and analysis of each model can help to improve the predictions of the thermal expansion behavior of the alloy under study. For multiphase composite materials, many models have been established and used to predict the CTE. Thus, we used four models to investigate the thermal behavior of bimetallic gradient alloys: linear rule of mixtures model (ROM), Kerner model, Schapery model, and Turner model. These four models are all based on the assumption that both the matrix and the reinforcement phase are uniform, and that only the linear elastic deformation occurs within a small volume strain [27]. In our system, the Si phase and the Al phase that contained Mg, Zn, and Cu formed a composited structure, and it is generally believed that the Si phase is the main factor affecting the CTE [18,20,24,26]. Therefore, for this bimetallic gradient alloy, the Al phase that contained Mg, Zn, and Cu is assumed as the matrix phase, and the Si phase is assumed as the reinforcement phase to calculate CTE in the prediction model for multiphase metal matrix composites. The linear rule of mixtures model can be given as [2]

(2) $$\alpha_{c}=\alpha_{m}V_{m}+\alpha_{p}V_{p}$$

where *α* represents the coefficient of linear thermal expansion, *V* represents the volume fraction, and subscripts *c*, *m*, and *p* represent the composite, matrix, and particle, respectively. In the formulae that follow, all physical quantities and symbols have the same meaning. The silicon content in the six samples was set to 0.03, 0.06, 0.09, 0.12, 0.15, and 0.18 as average values for each of them.

According to the Turner model, the deformation of the particles, matrix, and composite alloy as a whole was consistent and the shear deformation was negligible [16,29]. Only the uniform hydrostatic stresses that are not able to break the composite are considered in this model. It is worth noting that the angularity and distribution of reinforcement particles are ignored. Thus, the CTE given by the Turner model is calculated by the following formula:

(3) $$\alpha_{c}=\frac{\alpha_{m}K_{m}V_{m}+\alpha_{p}K_{p}V_{p}}{K_{m}V_{m}+K_{p}V_{p}}.$$

Based on the assumption of the Kerner model, the matrix evenly surrounds and wraps the spherical reinforcing phase [17,27]. In addition, the composite materials are isotropic and homogeneous. Compared with the previous model, the Kerner model considers both normal and shear stress together. The CTE for this model can be given by the following equation, in which the *G_m_* represents the shear modulus of the matrix:

(4) $$\alpha_{c}=\alpha_{m}V_{m}+\alpha_{p}V_{p}+V_{m}V_{p}\left(\alpha_{p}-\alpha_{m}\right)\times \frac{K_{p}-K_{m}}{V_{m}K_{m}+V_{p}K_{p}+\frac{3K_{m}K_{p}}{4G_{m}}}.$$

Since the Schapery model was developed on the basis of the Kerner model [19,29,30], the assumptions for the establishment of the model are also similar. For example, the matrix is considered isotropic and homogenous, evenly surrounding and wrapping the spherical reinforcing phase. Furthermore, the Poisson ratios of the composite alloy are consistent everywhere, as are the stress interactions between the reinforcement phase and the matrix. On the basis of Hashin’s bounds, under certain circumstances, only the upper and lower bounds of *K_c_* representing bulk modulus of the composite can be calculated by the following formulae [30]:

(5) $$K_{c}^{-}=K_{m}+V_{p}{\left[\frac{1}{K_{p}-K_{m}}+\frac{3\left(1-V_{p}\right)}{3K_{m}+4G_{m}}\right]}^{-1}$$

(6) $$K_{c}^{+}=K_{p}+\left(1-V_{p}\right){\left[\frac{1}{K_{m}-K_{p}}+\frac{3\left(1-V_{p}\right)}{3K_{p}+4G_{p}}\right]}^{-1}$$

where *G_m_* and *G_p_* represent the shear modulus of the matrix and particles, respectively. Then, the upper and lower boundaries of CTE in this model can be calculated by the following formulae.

Upper bound:

(7) $$\alpha_{c}^{+}=\alpha_{p}+\left(\alpha_{m}-\alpha_{p}\right)\times \frac{\frac{1}{K_{c}^{-}}-\frac{1}{K_{p}}}{\frac{1}{K_{m}}-\frac{1}{K_{p}}}.$$

Lower bound:

(8) $$\alpha_{c}^{-}=\alpha_{p}+\left(\alpha_{m}-\alpha_{p}\right)\times \frac{\frac{1}{K_{c}^{+}}-\frac{1}{K_{p}}}{\frac{1}{K_{m}}-\frac{1}{K_{p}}}.$$

Therefore, the Schapery model can only calculate the upper and lower boundaries of the CTE of the composite alloy; that is, it can only determine the range of the CTE. In addition, because of the similarity of the assumptions of the two models, the upper bound value calculated by the Schapery model is equivalent to the result calculated by the Kerner model [27].

**Figure 8:**
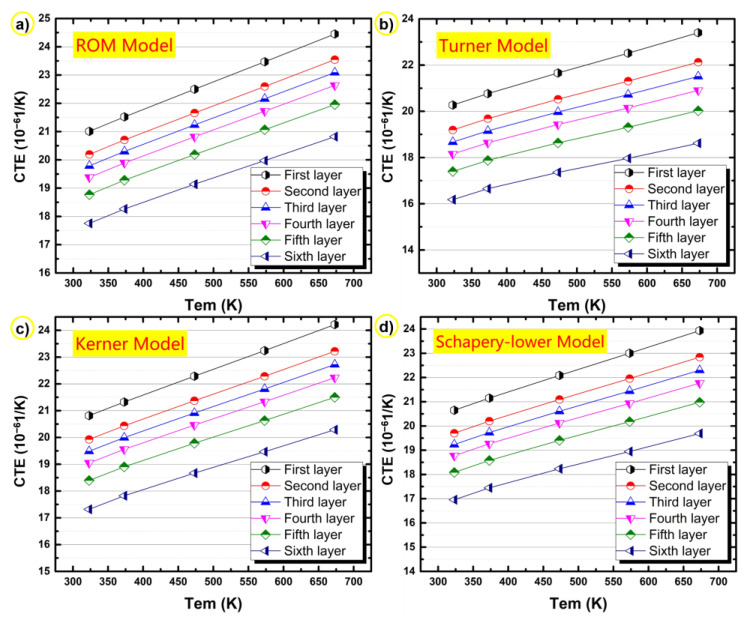
Calculated CTEs of the bimetallic gradient alloy in different layers using the (**a**) ROM model, (**b**) Turner model, (**c**) Kerner model, and (**d**) Schapery lower model.

Figure 8 shows the CTEs calculated according to the above different prediction models at different layers of the bimetallic gradient alloy. For these four theoretical models, it is certain that the calculated CTE values decrease with increasing layer number, and the CTE value of the sixth layer in the alloy is the lowest, which is consistent with the actual test results. The content of silicon increases with increasing layer number (see Figure 5a), leading to decreased CTE values.

**Figure 9:**
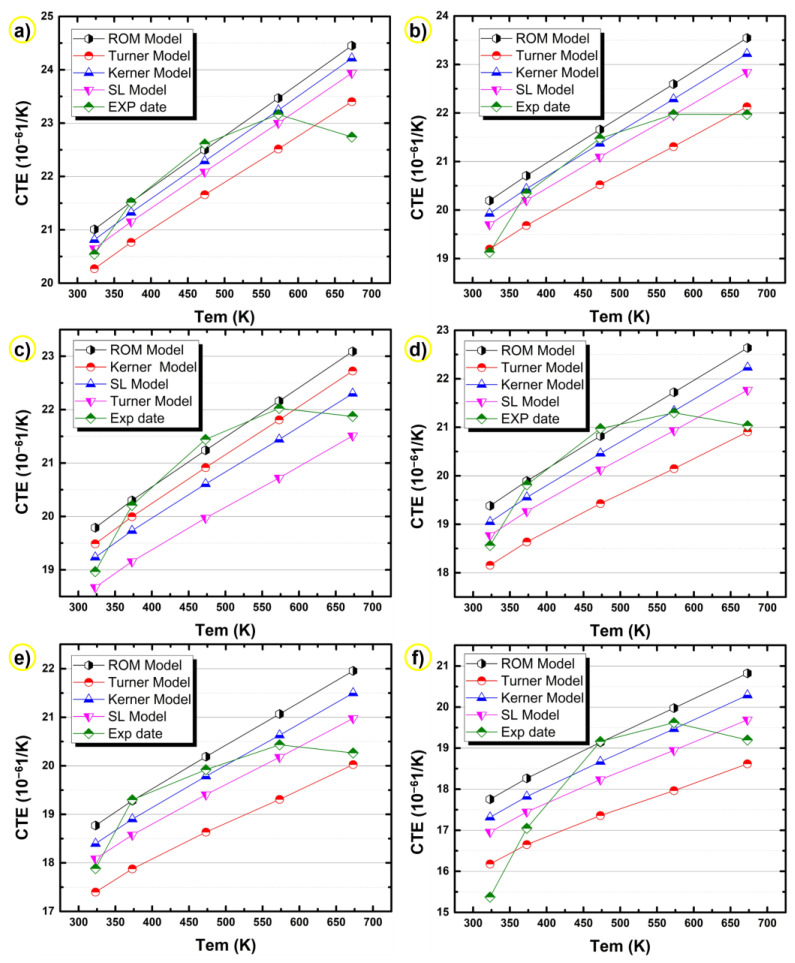
Comparison between theoretical and experimental CTEs for the bimetallic gradient alloy in different layers: (**a**) first layer, (**b**) second layer, (**c**) third layer, (**d**) fourth layer, (**e**) fifth layer, and (**f**) sixth layer.

As shown in Figure 9, comparing the actual test values of each layer of the alloy with the calculated values of the theoretical models, it is obvious that the theoretical curves of each layer are generally consistent with the experimental curves, and that the CTE increases with temperature. After comparing the curves in Figure 9, it can be seen that the ROM model estimates the highest CTEs and the Turner mode predicts the lowest CTEs. The CTEs calculated by the Kerner model and the SL model are located in the range where the calculated values of the ROM model are the upper boundary and the calculated values of the Turner model are the lower boundary. The experimental CTEs almost always fell in this range. When the temperature was low (≤323 K), the actual test CTEs were mainly between the values calculated by the Schapery lower model and the Turner model. When the temperature was greater than 373 K and less than 573 K, the curve formed by the actual test CTE was above the curve calculated by the Kerner model but below the curve calculated by the ROM model, which indicates that the CTE of the alloy was relatively close to the theoretical value calculated by these two models in this temperature range. In other words, the Kerner model and the ROM model are probably the most suitable for estimating the CTE of the present Al-20Si/7075 bimetallic gradient alloy within the range of temperature between 373 and 573 K.

When the temperature exceeds 573 K, the CTEs of all layers decreased with increasing temperature. In this temperature range, while the predictions of the four theoretical calculation models kept increasing, some regions of experimental curves were still within the range predicted by the theoretical models. As for the CTE at 673 K, they are basically close to the calculated values of the Turner model. It can be inferred that the four models are still useful for predicting the CTE of the present samples within the range of 350–650 K. Thus, in general, the four theoretical calculation models are still applicable in the present case. However, it is a challenge to determine which model performed the best at this temperature range because none of the four models fully covered the thermal expansion of practical composite materials.

The main reason for this difference is that the influencing factors considered in the two cases are different. In practice, thermal expansion is affected by many factors, such as microstructure evolution, thermal stress, silicon precipitation, and pores. As shown in Figure 5c,d, there are both small spherical silicon phases and coarse lath-shaped silicon phases in the samples. The small spherical silicon phase inhibits the matrix phase expansion, while the coarse silicon phase would increase the CTE. Furthermore, the sharp corners of coarse lath-shaped silicon particles cause more complex stresses, resulting in the discrepancy between the tested CTE and the predicted CTE. Due to the rapid solidification during the spray forming process, the silicon particles with fine microstructures and a uniform size distributed in the matrix could be obtained [31,32,33]. During the densification process, the present bimetallic gradient alloy samples were reheated and held for a period, and some of the silicon phase grew into a slightly irregular shape, as shown in Figure 5d. As a result of such thermal effects, the solid solution containing supersaturated silicon underwent a series of changes; for example, silicon atoms diffused and precipitated out, and the microstructure entered an equilibrium state as the primary silicon particles grew further. Zhu et al. found that the activation energy difference caused by different sizes of particles could also lead to the growth of particles [34]. They reported that the energy barrier for small particles to aggregate into large particles is reduced by the thermal effects that reduce the activation energy of the solute atom on the basis of the Gibbs–Thomas theory.

From the previous analysis, it can be found that during the heating process of the alloy, silicon phases are precipitated from the solid solution, the strength of the alloy decreases, the thermal stress of the alloy begins to be greater than the yield strength, and plastic deformation occurs, leading to the increase of CTE. In the early stage, plastic deformation plays a leading role in the rise of CTE. As the temperature gradually increases, the silicon precipitated gradually increases. Since aluminum and silicon have a certain offset effect on the plastic deformation, the growth rate of CTE will decrease. When the temperature exceeds 573 K, silicon phases are dissolved into the matrix again, and the strength of the material is greater than the thermal stress, leading to the decrease of CTE. The pores in the alloy found in the samples (Figure 5) expand due to heat during plastic deformation, and the CTE of the gas is relatively high, leading to the increase of the CTE of the gradient alloy.

For the theoretical model, all models are derived under the condition that only the strengthening effect of the particles on the composite material is considered, while the solid solution and particle precipitation are not taken into account. Although only the influence of strengthening phase was considered, resulting in differences between the actual test results and the theoretical prediction results, most of the test values were within the theoretical prediction range. Therefore, the four conventional theoretical models for composite materials (i.e., ROM model, Turner model, Kernel model, and Schapery lower model) were applicable to predict the CTE of the studied Al-20Si/7075 bimetallic gradient alloy.

## 4. Conclusions

In the present work, the thermal expansion behavior of Al-20Si/7075 bimetallic gradient alloy prepared by a co-spray forming process was investigated in detail. By testing the CTE of gradient alloy at different layers and using four different theoretical models of CTE of multiphase composite alloy, the following conclusions can be drawn:

1. The silicon content of Al-20Si/7075 bimetallic gradient alloy presents a gradient distribution in the direction of height, and correspondingly, the microstructure and CTE at different heights show the same distribution trend as that of silicon content.
2. The variation trend of CTE at different heights is consistent. As the temperature increased to 673 K, CTE first increased and then began to decrease when the temperature rose to 573 K.
3. The CTE of Al-20Si/7075 bimetallic gradient alloy is affected by many factors such as microstructure evolution, thermal stress, silicon precipitation, and pores. The small spherical silicon phase is beneficial to reduce CTE, while the coarse silicon phase would increase the CTE. In the process of heating, the silicon phase precipitates from the matrix, so the strength of the alloy is lower than the thermal stress, causing plastic deformation, and finally leading to the increase of CTE. CTE would decrease as the solid solubility of silicon in aluminum increases. The presence of primary pores in the alloy contributes to the increase of CTE.
4. Compared with the actual CTE showing a tendency of first increasing and then decreasing, the curves of four conventional theoretical models for composite materials (i.e., ROM model, Turner model, Kernel model, and Schapery lower model) show a linear relationship, which is different from the actual test results. The reason for this discrepancy is that the theoretical model only considers the strengthening effect of particles on composites.

## Figures and Tables

**Table 1:** Chemical composition of 7075 alloy (wt %).

Element	Zn	Mg	Cu	Fe	Si	Mn	Al
Content	5.63	2.21	1.41	0.26	0.1	0.05	Bal.

## Data Availability

The raw/processed data required to reproduce these findings cannot be shared at this time as the data also forms part of an ongoing study.
